# Exploring the Determinants of Patient Experiences Using the Digital Topic Modeling Approach

**DOI:** 10.1155/jonm/8183250

**Published:** 2025-07-11

**Authors:** Xiaofan Yu, Huanhuan Huang, Kexin Lin, Huan Wang, Shuangjiang Zheng, Xu Ran, Yang Liu, Hao Wu

**Affiliations:** ^1^Department of Medical Affairs Administration, The Southwest Hospital of Army Medical University, Chongqing 400038, China; ^2^The Design Academy, Sichuan Fine Arts Institute, Chongqing 400053, China; ^3^Department of Nursing, The First Affiliated Hospital of Chongqing Medical University, Chongqing 400016, China; ^4^Translational Medicine Center, The First Affiliated Hospital of Chongqing Medical University, Chongqing 400016, China; ^5^School of Business, Shandong Normal University, Jinan 250014, Shandong, China; ^6^Department of Development and Planning, Chongqing Medical University, Chongqing 400016, China; ^7^Department of Medical Affairs, The First Affiliated Hospital of Chongqing Medical University, Chongqing 400016, China; ^8^Doctor-Patient Experience Research Base, National Health Commission of the People's Republic of China, Beijing 100044, China

**Keywords:** digital topic modeling, patient experience, patient-centered care, service improvement

## Abstract

**Background:** As the healthcare landscape progressively adopts a patient-centered paradigm, the imperative to enhance patient experience has become more pronounced. Efforts to improve patient experience have yielded modest results, partly due to limited understanding of the key factors influencing patient expectations.

**Objective:** To explore the determinants of patient experiences through analyzing patient feedbacks, assisting healthcare institutions in prioritizing service improvements.

**Methods:** A digital topic modeling approach was employed. Data were derived from a secondary analysis of the National Patient Experience Base, incorporating patient feedback from 226 hospitals. Initially, the feedback text data underwent a cleansing process, and the sentiment intensity within the text was quantified using the SnowNLP algorithm, and XGBoost classifier was utilized to categorize sentiments as positive or negative. Subsequently, the feedbacks were subjected to topic clustering using the BERT model and X-means clustering algorithm. Third, TextRank was applied to extract significant keywords from each cluster, and these keywords were analyzed to identify the determinants that impact patient experience.

**Results:** A total of 4689 patients' feedbacks were collected, comprising 2918 outpatients and 1771 inpatients from 165 tertiary and 61 secondary hospitals across 24 provinces. Through cluster analysis, 10 main clusters emerged (two of which were positive response and eight were negative response). By qualitatively synthesizing, patient experiences were distilled into five determinants: treatment, service, environment, economic, and process.

**Conclusions:** The findings underscore the importance of a holistic approach to patient experience enhancement, where healthcare providers must address not only the clinical aspects of care but also the service delivery, environmental conditions, economic considerations, and procedural efficiency. By identifying and prioritizing the improvement of these determinants, healthcare organizations can tailor their services to better meet patient expectations and enhance overall satisfaction.

## 1. Introduction

Patient experience is a complex construct, encapsulating emotional, cognitive, and behavioral facets [1], extending beyond mere satisfaction with care [2]. It includes all interactions between healthcare institutions and patients throughout the continuum of care, from scheduling appointments, admission, treatment, to discharge [3]. Existing research substantiates that an enhanced patient experience significantly bolsters patients' trust in healthcare institutions and individual practitioners [4], cultivates harmonious doctor–patient relationships [5], and contributes to the development of a sustainable systems [6]. In addition, research indicates that a positive patient experience has a beneficial impact on clinical effectiveness and patient safety [7], thereby cutting costs [8].

As patient-centered care becomes more prominent in the healthcare landscape, patient experience has established itself as a cornerstone of healthcare service quality [9]. This consensus has driven exploration into the impact of patient experience and perceptions over the past decades [10]. For instance, the NHS Patient Survey Program in the UK [11] and the Consumer Assessment of Healthcare Providers and Systems Hospital Survey in the US [12] have provided tools and empirical quantitative evidence for measuring patient experience. In China, research and practical work on improving patient experience have also been conducted [13]. Notably, the Chinese government issued a policy in May 2025 aimed at implementing a nationwide thematic program (2023–2025) to improve the medical experience and enhance patient satisfaction [14]. Despite these efforts, the results have been modest, partly due to a lack of precise insight into factors significantly affecting patient expectations.

Research on the determinants of patient experience predominantly employs patient self-reports through surveys [15, 16], which mainly rely on structured questionnaires. However, studies have found that although there are as many as 23 available tools, only a limited number of studies possess methodologically sound designs, impacting the credibility of these tools [17]. The other typical approach employs interviews, which is advantageous in identifying specific populations such as patients with cancer [18, 19] or ethnic minorities [20]. While in practice, free-text responses are often underutilized [21]. Thus, although traditional methods such as surveys and interviews provide valuable insights into patient experiences, a pressing need for innovative methodologies to capture the complexity of patient feedback.

In this context, feedback represents an individual's assessment, judgment, and evaluation of events or ideas [22]. Such feedback is distinguished by its low cost, ease of access, timeliness, and voluntary nature, which can compensate for the deficiencies of these traditional data collection methods [23]. Moreover, this feedback reflects the genuine emotions and opinions of patients, providing healthcare providers with essential insights to identify specific aspects of service performance needing improvement [24].

With the evolution of Natural Language Processing (NLP) technology, it has become possible to mine emotional tendencies and valuable determinants from patient feedbacks [25], potentially serving as a rich data source for exploring the determinants of patient experience. Digital topic modeling is a technique in NLP, fundamentally grounded in text mining [26]. It is designed to uncover hidden semantic patterns within a corpus of text and automatically discern the inherent topics. This method leverages unsupervised machine learning algorithms for textual data analysis, identifying and clustering groups of similar words, thus exposing the thematic structures embedded within the texts [27]. This data-driven approach reduces human bias and allows for the simultaneous consideration of multiple variables, thus achieving a more comprehensive analysis of the factors influencing patient experience [28]. It has previously been applied in the study of public emotions in social media [29].

Therefore, this study aims to use patients' feedbacks as a data source and employs digital topic modeling to identify the determinants of patient experience, intending to provide targeted strategies for healthcare institutions to meet patient expectations and promote continuous service improvement.

## 2. Methods

### 2.1. Study Design

**Figure 1:**
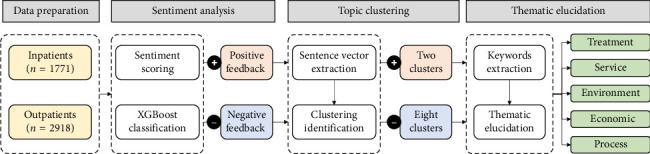
Study design.

Referring to previous studies [30], our research methodology encompassed the following four steps: (1) data preparation: patient feedback regarding their medical experiences was systematically collected from both outpatient and inpatient sources. The gathered data underwent a stringent cleaning and preprocessing phase to ensure data integrity and consistency. (2) Sentiment analysis: the SnowNLP algorithm was employed to calculate sentiment scores, and the XGBoost classifier was utilized to categorize the text into positive and negative categorization. (3) Topic clustering: both positive and negative comments were subjected to sentence vector extraction and clustering identification to discern patterns and groupings within the data. (4) Thematic elucidation: TextRank was applied to identify key terms within each cluster. These terms were then qualitatively synthesized to recognize overarching themes. These steps are illustrated in Figure 1.

### 2.2. Data Preparation

Data for this study were extracted from the secondary analysis of the Doctor–Patient Experience Research Base, conducted by the National Health Commission of the People's Republic of China, encompassing data from 226 hospitals. Patients aged 18 years or older were included. For feedbacks processing, the *Jieba* Python library was utilized. The data preprocessing phase involved a systematic cleansing process where non-Unicode characters, extraneous whitespace, and specific symbols were identified and removed using regular expression techniques. Furthermore, to enhance the analytical value, numeric entities, stop words, and common terms with limited relevance such as “doctor,” “hospital,” and “patient” were excluded from the analysis. This methodological approach is consistent with prevailing standards in the academic literature [31, 32].

### 2.3. Sentiment Analysis

Sentiment analysis, a key task in NLP, aims to detect subjective opinions and classify emotions in textual data [33]. Among Python libraries tailored for NLP, SnowNLP specializes in Chinese text processing and is widely used for its efficiency in sentiment analysis [34]. To enhance classification performance, this study employs XGBoost, a gradient-boosting algorithm known for its high accuracy in handling structured text features. SnowNLP employs machine learning models trained on annotated datasets to predict sentiment polarity, with scores ranging from 0 to 1. Specifically, when the score exceeds 0.5, the sentiment is classified as more positive, and scores closer to 1 reflect heightened positivity. Conversely, scores below 0.5 denote more negative sentiment, with values nearing 0 indicating greater negativity. SnowNLP's accuracy has been validated particularly for online reviews, making it a practical tool for Chinese sentiment analysis [35].

### 2.4. Topic Clustering

Patient feedback on healthcare experiences often contains complex sentiments and diverse opinions. To analyze these unstructured texts, this study adopts a two-stage approach. Initially, the Bidirectional Encoder Representations from Transformers (BERTs) model, which performs well in sentiment analysis [36], was utilized to transform textual data into high-dimensional semantic vectors, capturing the intricacies and semantic relationships within the text. Subsequently, the X-means clustering algorithm was employed to categorize sentences into thematic clusters based on the proximity of their vector representations [37]. This unsupervised learning technique capitalized on the similarity between vectors, measured using metrics such as Euclidean distance or cosine similarity, to aggregate semantically similar sentences into distinct clusters [38]. This unsupervised approach circumvents the need for annotated training data while effectively identifying emergent themes in patient feedback, consistent with methodologies employed in clinical sentiment analysis [39, 40].

### 2.5. Thematic Elucidation

The *TextRank* algorithm, an unsupervised learning approach, adeptly discerns semantic relationships among terms in text based on graph ranking theory [41]. Following the extraction of keywords by *TextRank*, a thorough qualitative analysis was executed to systematize these keywords into meaningful themes. This methodology included a detailed manual review of the extracted terms, identification of thematic patterns, and the clustering of keywords into overarching themes that represent the multifaceted dimensions of patient experience. The final determination of themes was collaboratively conducted by two researchers (K. L. and H. H.), both with relevant field experience and knowledge. In cases of disagreement, a third scholar (H. W.) served as an arbitrator to reach a consensus, thereby forming the definitive themes.

### 2.6. Ethical Considerations

Data were sourced from anonymized documents, ensuring patient privacy. With no interventions involved, the study posed minimal risk. Adhering to the Declaration of Helsinki, the secondary data analysis was approved by the Ethics Committee of the First Affiliated Hospital of Chongqing Medical University (Approval no.: 2024.203.02), safeguarding participant privacy and data confidentiality.

## 3. Results

### 3.1. Description Analysis

A total of 4689 patients from 226 hospitals—165 of which were tertiary hospitals and 61 were secondary hospitals—located across 24 provinces were ultimately included in the study. Of these, 2918 were outpatients and 1771 were inpatients. The gender distribution was relatively balanced, with females comprising 48.24% and males 51.76% of the total sample. The age distribution was skewed toward younger adults, with 55.81% of the total sample falling within the 18–39 years age bracket. Most patients resided within the city/district of the province, indicating a predominantly local patient base. Furthermore, 43.02% of the patients were covered by urban employee medical insurance. Family income varied widely among patients, with the largest group earning less than 3000 RMB, accounting for 43.08% of the sample. In terms of the primary reasons for choosing the hospital, hospital reputation was the most influential factor, cited by 34.51% of the patients, followed by medical expertise at 25.25%. Details are presented in Table 1.

### 3.2. Sentiment Analysis and Clustering

**Figure 2:**
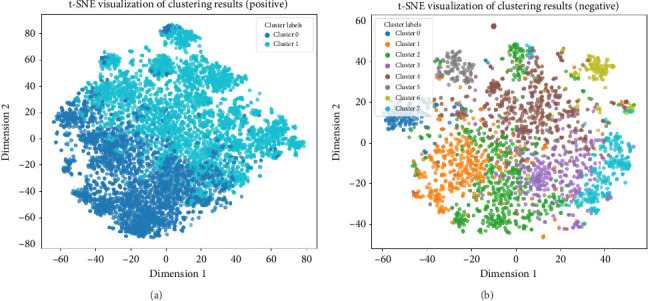
t-SNE visualization of clustered patient feedback in both positive (a) and negative (b) response categories.

For positive feedback, this study identified two main themes; for negative feedback, eight themes were established (as seen in Figure 2). Postclustering, the feedback was categorized into 10 distinct clusters. Examples of pivotal terms from each cluster are presented in Table 2, offering insights into the specific issues and sentiments expressed by patients.

### 3.3. Thematic Elucidation

**Figure 3:**
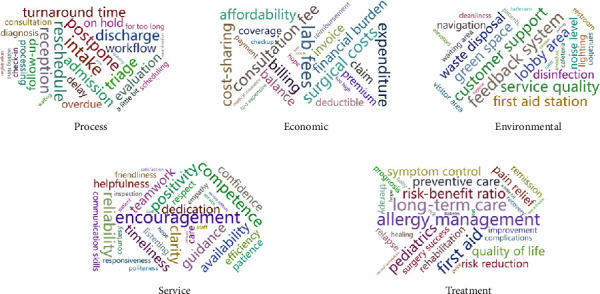
Word cloud visualization of subdimensions in patient experience assessment.

Upon thematic elucidation, the determinants of patient experience were categorized into five subthemes, as delineated in Table 3 and Figure 3. This table illustrates the subthemes of patient experience, identified through group discussion, along with representative keywords and exemplary patient feedback for each subaccount.

## 4. Discussion

This study utilizes data-driven models, particularly digital content analysis, to elucidate the key factors influencing patient experiences in China. This analytical approach offers a novel perspective for understanding patient experiences, reflecting the multidimensional nature of patient feedback and providing valuable insights for future improvements in patient care.

Our findings reveal a pronounced negativity bias in patient feedback, with negative sentiment clusters significantly outnumbering positive ones. This aligns with prior research (82.81% of the analyzed posts) [42] and is consistent with the negativity bias theory [43], which posits that adverse experiences exert a disproportionately strong psychological impact, prompting patients to vocalize dissatisfaction more readily than satisfaction. These results underscore the need for healthcare administrators to shift from reactive complaint resolution to proactive mitigation strategies—such as implementing real-time sentiment monitoring systems and rapid-response patient advocacy teams—to preemptively address systemic pain points.

Our study identified a broad range of factors influencing patient experience, which were categorized into five key subthemes: treatment-related factors, service quality, environmental factors, process-related aspects, and economic considerations. These findings align with prior research [44]. Consistent with a meta-analysis [45], our results indicate that treatment and service quality are nearly universal themes across patient experience studies. This phenomenon can be explained by several theoretical frameworks. According to Maslow's hierarchy of needs, the primary purpose of seeking medical care is to alleviate suffering or preserve life, making treatment efficacy the fundamental determinant of patient experience. If treatment outcomes are poor (e.g., surgical failure), satisfaction in other dimensions, such as environmental comfort, may still be negatively impacted—reflecting the “basic factors” principle in the Kano model [46].

In addition, recent research underscores that “being treated with respect and dignity” is integral to patient experience, a dynamic construct encompassing interactions, emotional responses, and the healthcare journey [47]. This is shaped by trust in healthcare professionals, nurse staffing levels, and communication practices [48–50]. Patients in medical settings are often in a vulnerable state, making them highly sensitive to the attitudes of healthcare providers (e.g., respect and empathy). This rule suggests to managers, services cannot replace technology, but available services can amplify the value of technology.

Similar to prior empirical studies [51–53], environmental factors can simultaneously affect patients' physical, cognitive, and emotional systems [54]. For instance, noise levels exceeding 45 dB can elevate muscle cortisol levels, delaying wound healing, whereas natural light helps regulate melatonin secretion and reduces postoperative delirium. Thus, care environments should address both clinical requirements and the diverse needs of patient populations, particularly vulnerable groups [55], such as patients receive palliative and end of life care [56].

Existing research also highlights the critical role of process efficiency (e.g., waiting times and referral processes) in shaping patient experience. Liao's investigation reveals there are always extensive waiting times in clinics [57], while the journey is not fulfilling and enjoyable [58], especially when patients, as a vulnerable group, are suffering physical discomfort and health uncertainty. This situation can even lead to feelings of powerlessness, indignity, and humiliation [59, 60]. Notably, waiting times may be subjectively amplified due to physical pain. Therefore, we recommend that healthcare managers employ service design thinking to restructure processes, optimizing workflows based on patient journey mapping [61].

Unlike previous studies, our research identified economic factors as an independent subtheme. In some high-income countries, these may be categorized under “service accessibility,” reflecting differences in healthcare payment systems [62]. This distinction may be attributed to our study population, where 10.73% (*n* = 503) of the respondents were self-paying patients and 43.08% (*n* = 2020) reported a monthly household income below 3000 RMB. This suggests that a significant proportion of patients have limited financial capacity to bear medical expenses. Although China has made substantial progress in poverty alleviation and healthcare system reforms (e.g., critical illness insurance and medical assistance programs), the phenomena of “poverty due to illness” or “returning to poverty due to illness” have not been entirely eradicated [63]. Thus, healthcare managers should enhance cost transparency, reduce patient anxiety, and provide additional support and explanations for low-income patients.

A key strength of our study is its large-scale, nationally representative sample. In addition, the use of data-driven techniques is another notable strength. These methods prove particularly valuable in the face of challenges such as research bias, difficulties in respondent recruitment, complexity of results, and operational costs [64]. Although direct performance comparisons with baseline models are not presented, our methodology aligns with proven paradigms [65–67]. The extracted themes further resonate with prior surveys, indirectly validating our results. Future work should expand comparisons, especially for emerging contexts like internet hospitals [68].

## 5. Conclusions

This study provides a novel approach to understanding patient experiences by applying data-driven models. The identification of key influencing factors and their categorization into distinct determinants offers a framework for targeted interventions aimed at improving patient satisfaction and overall healthcare quality.

## Figures and Tables

**Table 1:** General information of the participants (*N* = 4689).

Variables	Total (*N* = 4689)	Outpatients (*N* = 2918)	Inpatients (*N* = 1771)
*Gender*			
Female	2262 (48.24%)	1504 (51.54%)	758 (42.80%)
Male	2427 (51.76%)	1414 (48.46%)	1013 (57.20%)

*Age (years)*			
18–39	2617 (55.81%)	1938 (66.42%)	679 (38.34%)
40–59	1093 (23.31%)	613 (21.01%)	480 (27.10%)
60–79	840 (17.91%)	327 (11.21%)	513 (28.97%)
≥ 80	139 (2.96%)	40 (1.37%)	99 (5.59%)

*Residence status*			
City/district in province	3834 (81.77%)	2371 (81.25%)	1463 (82.61%)
Other cities in province	438 (9.34%)	308 (10.56%)	130 (7.34%)
Out-of-province	399 (8.51%)	225 (7.71%)	174 (9.82%)
Other	18 (0.38%)	14 (0.48%)	4 (0.23%)

*Insurance type*			
Resident medical insurance	1786 (38.09%)	1121 (38.42%)	665 (37.55%)
Urban employee medical insurance	2017 (43.02%)	1116 (38.25%)	901 (50.88%)
Out-of-pocket	503 (10.73%)	435 (14.91%)	68 (3.84%)
Full reimbursement	157 (3.35%)	124 (4.25%)	33 (1.86%)
Other	226 (4.82%)	122 (4.18%)	104 (5.87%)

*Family income (¥)*			
< 3k	2020 (43.08%)	1138 (39.00%)	882 (49.80%)
3–10k	1649 (35.17%)	1022 (35.02%)	627 (35.40%)
10–20k	644 (13.73%)	491 (16.83%)	153 (8.64%)
20–50k	253 (5.40%)	183 (6.27%)	70 (3.95%)
≥ 50k	123 (2.62%)	84 (2.88%)	39 (2.20%)

*Primary reason for choosing hospital*			
Hospital reputation	1618 (34.51%)	1003 (34.37%)	615 (34.73%)
Medical expertise	1184 (25.25%)	653 (22.38%)	531 (29.98%)
Geographic convenience	967 (20.62%)	694 (23.78%)	273 (15.42%)
Service attitude	396 (8.45%)	186 (6.37%)	210 (11.86%)
Personal relationship and trust	164 (3.50%)	116 (3.98%)	48 (2.71%)
Hospital environment	123 (2.62%)	87 (2.98%)	36 (2.03%)
Economic factors	34 (0.73%)	22 (0.75%)	12 (0.68%)
Other	203 (4.33%)	157 (5.38%)	46 (2.60%)

**Table 2:** The top 10 keywords of each cluster.

Categorization	Cluster	Keywords (weights)
Positive	0	Registration (0.054), examination (0.049), queuing (0.044), time (0.035), patient (0.023)
	1	A bit (0.069), hope (0.043), appointment (0.036), project (0.021), increase (0.02)

Negative	2	Time (0.206), waiting (0.098), examination (0.038), too long (0.032), queuing (0.03)
	3	Hope (0.064), related (0.028), examination (0.026), family members (0.019), medical report (0.016)
	4	Professional (0.041), guidance (0.041), staff (0.032), parking (0.026), trouble (0.025)
	5	Attitude (0.095), not good (0.046), medical staff (0.03), canteen (0.029), patient (0.026)
	6	Increase (0.055), parking spots (0.048), tension (0.043), hope (0.031), queue jumping (0.025)
	7	Fees (0.059), hope (0.03), restrooms (0.029), expansion (0.028), too expensive (0.027)
	8	Communication (0.066), negative (0.029), experience (0.027), service attitude (0.025), dining (0.021)
	9	Hygiene (0.047), patients (0.045), toilets (0.036), medical care (0.032), improvement (0.021)

**Table 3:** The subaccounts of patient experience identified through group discussion.

Subaccounts	Cluster	Keywords	Exemplar patient feedback
Process	2	Time, queuing, examination, registration, waiting, waiting time is too long, to make an appointment, a little bit, for too long	Could you ensure that the medication for our critical care patients, particularly those with cardiovascular and cerebrovascular diseases, is distributed in the morning? If it is only dispensed in the afternoon or at the end of the day, it would be quite unbearable for us.
Economic	7	Fees, costs, medical insurance, too high, too expensive, check-up, hope, reimbursement, payment, out-of-pocket	The cost of medical examinations is excessively high.
Environmental	9, 6	Parking, canteen, hygiene, toilet, bathroom, parking space, improve, elevators, patients	The food in the canteen is unpalatable; I have lost several pounds in just a few days.
Service	8, 3, 5	Attitude, service attitude, bad, satisfaction, service, patient, and staff, hope, and inspection	They (doctors) are diligent and responsible in their work, and show great warmth toward patients.
Treatment	0, 1, 4	Cervical spondylosis, see a doctor, diabetes, a little bit, see a doctor, cut, hope, price, risk, baby	A month postsurgery, the testicles have settled nicely in the scrotum, the incision is tiny and pretty much gone, and the healing is looking great.

## Data Availability

The data that support the findings of this study are available from the corresponding author upon reasonable request.
